# The structural equation modeling of personal aspects, environmental aspects, and happiness among older adults living alone: a cross-sectional study

**DOI:** 10.1186/s12877-021-02430-4

**Published:** 2021-09-04

**Authors:** Eun Jeong Hwang, In Ok Sim

**Affiliations:** 1 Department of Nursing, Sehan University, 1113 Noksaek-ro, Samho-eup, Yeongam-gun, Jeollanam-do 58447 Republic of Korea; 2 Red Cross College of Nursing, Chung-Ang University, 84 Heukseok-ro, Dongjak-gu, Seoul, 06974 Republic of Korea

**Keywords:** Structural equation modeling, Social, Environment, Happiness, Living alone

## Abstract

**Background:**

The happiness of older adults living alone warrants attention because they are more vulnerable to unhappiness than those living with families. The present study aimed to construct and test a structural equation model to elucidate the relationship among participation in social activities, satisfaction with the neighborhood environment, subjective health status, and happiness in older adults living alone in South Korea.

**Methods:**

Secondary data of 2768 older adults (605 males and 2163 females) living on their own were extracted from the 2017 Korean Community Health Survey and used in this cross-sectional study. Data were collected via self-reported questionnaires and analyzed using SPSS version 20.0 and AMOS version 20.0.

**Results:**

The hypothetical model exhibited a good fit: χ^2^ = 342.06 (df = 58, *p <* .001), goodness-of-fit index = .98, adjected goodness-of-fit index = .97, root mean square error of approximation = .04, and nonstandard fit index = .92. Participation in social activities had a significant effect on participants’ subjective health status (path coefficient = .45, *p* = .001) and happiness (path coefficient = .20, *p* = .003).

**Conclusions:**

Interventions to improve the health and happiness of older adults living alone should aim to enhance their social and physical environmental dimensions based on the participants’ various social activities and their neighborhoods’ characteristics.

## Background

In 2017, South Korea officially became an aging society when the proportion of people aged ≥65 years reached 14.2% of the total population. Furthermore, the proportion of older adults living alone showed 24.4% of the total one-person households in 2017 [1].

Some studies have suggested that older adults living alone not only experience depression, poorer health, less social capital, loneliness, increased prevalence of social isolation, reduced social support, lower quality of life, and less subjective happiness than those living with others, but they are also more likely to feel sad, hopeless, and worthless [2–4]. Conversely, many older adults value the independence, familiarity, personal comfort, and privacy of living alone and age just as successfully or even better than those who co-reside [5, 6].

The happiness of older adults living alone warrants attention because they are more vulnerable to the adverse effects of social isolation than older adults living with their families due to greater risk of losing their spouses and friends, which at the same time makes them more dependent on available social capital. Happiness is a broad concept that includes various related concepts, such as life satisfaction, a good life, a better life, well-being, and quality of life [7–13]. Happiness is a broad concept encompassing happy life; quality of life is an objective condition, and happy emotions are subjective evaluations. Various factors, including depressive symptoms, health status, social support, and social network, have been understood to affect the happiness of older adults living alone [5, 7]. Most studies on the happiness of older adults living alone limited their focus to the influence of health and social support, although some authors adopted a broader approach. For instance, Kim [9] suggested that household income, depression, subjective stress levels, subjective health levels, quality of life, and lack of required medical services influence the happiness of adults aged 65 years and over and living alone. Van Leeuwen and colleagues [14] identified nine quality-of-life domains for older adults (mean age 71–91 years) living alone; these include autonomy, role and activity, health perception, relationships, attitude and adaptation, emotional comfort, spirituality, home and neighborhood, and financial security. The World Health Organization [15] identified health and social services, behavioral determinants, personal determinants, physical environment, social determinants, and economic determinants as factors affecting happy and active aging [16]. Such findings suggest that social participation, housing environment, and health status are the major factors influencing happiness in older adults living alone. In listing the factors influencing happiness in older adults, it is also important to understand the structural relevance of these factors.

Older adults’ participation in social activities is an essential element of successful aging [16]. Through their participation in social activities, older adults can fulfill their appropriate social roles, form friendly relationships, engage in productive activities, prevent loneliness, and increase happiness [17]. Kim and Ha [17] suggested that sports, volunteer activities, and travel are productive leisure activities that enhance physical health and reduce suicidal thoughts in older adults living alone. The idea that the health and well-being of individuals who reside in specific environments for prolonged periods are influenced by their neighborhood environment, regardless of their personal characteristics, is one of the most widely tested hypotheses [18, 19]. Older adults spend most of their time without any specific purpose in the community; therefore, they might be affected by their local environment and surrounding social networks [20]. Stahl and colleagues [19] reported that a social community network based on traditional relationships between neighbors used as a poor alternative support system for older adults living alone contributed to solving their various difficulties. Furthermore, Putrik and colleagues [18] found that the neighborhood characteristics influenced the subjective health and depression levels of residents. They divided neighborhood characteristics into physical (e.g., buildings and public transport) and social (e.g., social ties, stability) characteristics.

Above all, older individuals wish to maintain good health during aging. Quality-of-life and happiness depend largely on health status; a disease or disability can affect independence by limiting individual behaviors [11]. Therefore, subjective health awareness is considered a major factor influencing happiness [21].

In general, it is desirable for older adults to achieve happiness by maintaining connections with the social community while receiving physical care, emotional support, and financial support from their families [22, 23]. However, older adults living alone are very vulnerable to experiencing a lack of support from their kinship community; therefore, obtaining support through social resources and non-kinship support systems may help them enjoy a healthy and happy life. To enhance successful aging, it is important to identify the multidimensional factors affecting happiness in older adults living alone and empirically identify the relationship between these factors and the health of older adults. Based on the relevant literature, the factors influencing the happiness of older adults living alone can be largely divided into personal and environmental aspects.

This study developed and tested a model to explain and predict happiness in older adults living alone in Korea and developed a hypothetical model for happiness in these individuals. In this regard, we propose a structural model that explains and predicts happiness in older adults living alone by verifying the fit of the hypothesized model. Finally, we investigated the direct, indirect, and total effects of factors affecting happiness in older adults living alone by evaluating participants’ subjective feelings regarding their health and their objective interactions with the environment.

## Methods

### Design

A cross-sectional study was conducted using the secondary data of 2768 older adults who participated in the 2017 Korean Community Health Survey (KCHS) conducted by the Korea Disease Control and Prevention Agency (KDCA). The survey collected data via self-reported questionnaires. An official request to the KDCA to use the data for our research was granted. The Institutional Review Board of Sehan University (approval number 2019–29) granted permission to conduct the study.

This study aimed to develop and test a structural equation model describing the structural relationship among personal aspects, environmental aspects, subjective health status, and happiness in older adults living alone in Korea. The personal aspects consist of participation in social, religious, and philanthropic activities. The environmental aspects consist of satisfaction with the physical neighborhood environment, the social neighborhood environment, and the condition of public services.

### Research model and hypothesis setting

**Fig. 1:**
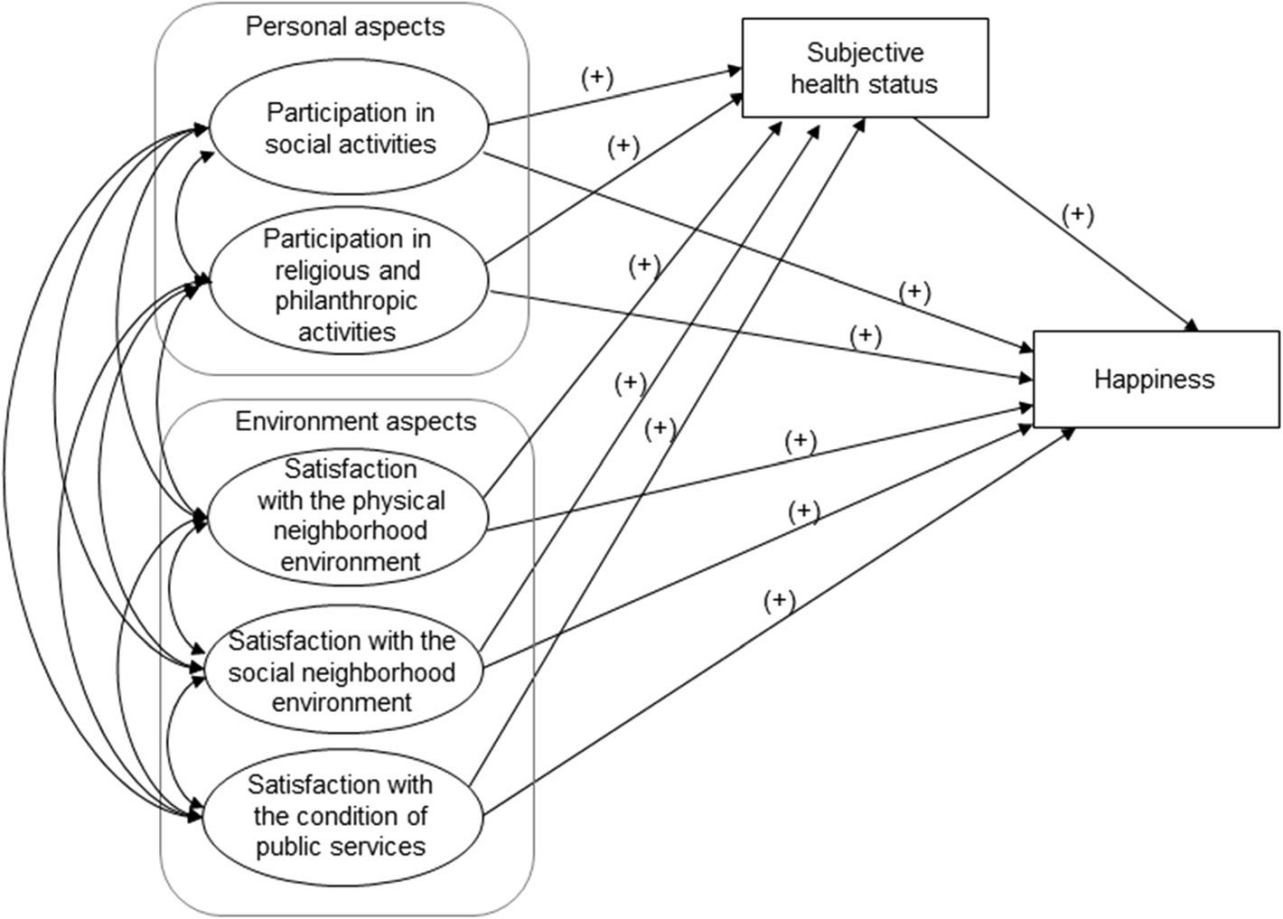
Hypothetical framework

We developed a hypothetical framework of personal and environmental factors affecting the health and happiness of older adults living alone based on a literature review, as shown in Fig. 1. This study’s hypothesis was proposed assuming five exogenous variables, one intervening variable, and one endogenous variable. The five exogenous variables were participation in social activities, participation in religious and philanthropic activities, satisfaction with the physical neighborhood environment, satisfaction with the social neighborhood environment, and satisfaction with the condition of public services. The endogenous variable was happiness. Subjective health status was considered an intervening variable that influences the relationship between the five exogenous variables and happiness. Thus, the present study analyzed the direct effects of the exogenous variables and the indirect effects of subjective health status on happiness in older adults living alone.

### Data collection and procedures

This study used the secondary data extracted from the 2017 KCHS conducted by the Korea Disease Control and Prevention Agency (KDCA). The government used these data to promote a comprehensive and multidimensional approach to support the happiness of older adults living alone. The data are available free of charge for research purposes. The data acquition and research procedures of this study were carried out in accordance with relevant guidelines and regulations. These annual surveys conducted nationwide utilize the public health center network. The sample for the 2017 survey comprises adults aged ≥19 years. All sample households were included in the survey. During data collection, trained researchers visited the selected households, explained the purpose of the survey to the respondents, ensured the confidentiality of their responses, and administered an electronic questionnaire through a one-to-one interview. The survey period was from August 16 to October 31, 2017. The present study utilized the 2017 KCHS data for 3008 individuals aged ≥65 years who lived alone at home. After excluding participants with missing data (*n =* 240), the analysis involved 2768 older adults.

### Exogenous variables

#### Participation in social activities

Participation in social activities among older adults living alone was a latent variable measured by two observational variables: “regular participation in social activities (gatherings with friends, alumni associations, senior citizens’ associations, fraternities, and social gatherings) at least once a month” and “regular participation in leisure activities at least once a month.” Each item was measured on a dichotomous scale (1 = non-participation and 2 = participation). The total score for participation in social activities was 4 points. This measure had a Kuder-Richardson formula 20 (KR-20) value of .60.

#### Participation in religious and philanthropic activities

Participation in religious and philanthropic activities among older adults living alone was a latent variable measured by two observational variables: “regular participation in religious activities at least once a month” and “regular participation in philanthropic activities at least once a month.” Each item was measured on a dichotomous scale (1 = non-participation and 2 = participation). The total score for participation in religious and philanthropic activities was 4 points. The reliability of this measure is evidenced by a KR-20 value of .60.

#### Satisfaction with the physical neighborhood environment

Satisfaction with the physical neighborhood environment among older adults living alone was a latent variable measured by three observational variables: “neighborhood safety level (disasters, traffic accidents, work accidents, and crime),” “natural environment (air and water quality),” and “living environment (electricity, water, sewage, garbage collection, and athletic facilities).” Each item was measured on a dichotomous scale (1 = dissatisfied and 2 = satisfied). The total score of satisfaction with the physical neighborhood environment was 6 points. The reliability of this measure is evidenced by a KR-20 value of .63.

#### Satisfaction with the social neighborhood environment

Satisfaction with the social neighborhood environment among older adults living alone was a latent variable measured by three observational variables: “trust in neighbors,” “contact with family (or relatives),” and “contact with neighbors. Each item was measured on a dichotomous scale (1 = dissatisfied and 2 = satisfied). The total score of satisfaction with the social neighborhood environment was 6 points. The reliability of this measure is evidenced by a KR-20 value of .60.

#### Satisfaction with the condition of public services

Satisfaction with the condition of public services among older adults living alone was a latent variable measured by two observational variables: “condition of public transport (buses, taxis, trains, and subway),” and “condition of medical facilities (hospitals, community health centers, oriental medicine, and pharmacies).” Each item was measured on a dichotomous scale (1 = dissatisfied and 2 = satisfied). The total score of satisfaction with the condition of public services was 4 points. The reliability of this measure is evidenced by a KR-20 value of .60.

### Endogenous variables

#### Subjective health status

The subjective health status of older adults living alone was an observational variable assessed by one question on participants’ health condition at the time of the survey. Participants indicated their state of health on a five-point Likert scale (1 = very bad, 2 = bad, 3 = neutral, 4 = good, and 5 = very good).

#### Happiness

The happiness of older adults living alone was an observational variable assessed by one question on participants’ happiness level at the time of the survey. Participants marked their happiness on a 10-point graphic rating scale (1 = terribly unhappy and 10 = extremely happy), with higher scores indicating higher happiness levels.

### Statistical analyses

Data were analyzed using SPSS version 20.0 for Windows and AMOS version 20.0. All variables, including participants’ general characteristics, were analyzed using descriptive statistics. The reliability of the measures assessing the exogenous variables was analyzed using KR-20 because it utilizes a dichotomous scale. Correlation and multicollinearity between observational variables were confirmed using Spearman’s rank correlation coefficient, the variance inflation factor (VIF), and tolerance. The normality of variables was confirmed through skewness and kurtosis; the following values were considered to indicate non-normality. A confirmatory factor analysis was performed to determine whether the observational variables that comprised each latent variable in the structural equation model were properly constructed. Structural equation modeling was used to verify the derived model and calculate the direct and indirect path coefficients of factors influencing the model. The χ^2^ test was used to confirm the goodness-of-fit of the structural model, which determined the completeness of the model and confirmed a good fit for the total population data. Additionally, the model’s goodness-of-fit was confirmed using the goodness-of-fit index (GFI), adjusted goodness-of-fit index (AGFI), root mean square error of approximation (RMSEA), nonstandard fit index (NFI), relative fit index (RFI), incremental fit index (IFI), Tucker-Lewis index (TLI), and comparative fit index (CFI). The significance of direct, indirect, and total effects was confirmed by bootstrapping.

## Results

### Sample characteristics

Table 1 summarizes the descriptive statistics for participants’ general characteristics and the endogenous and exogenous variables. The sample comprised 605 males (21.9%) and 2163 females (78.1%), and the mean age was 74.89 ± 6.43 years (range 65–100). Regarding the age category, the age group 75–79 years had the highest frequency with 719 participants (26.0%), and the age group ≥80 years had the lowest frequency with 666 participants (24.1%). Regarding the educational category, elementary school graduates had the highest frequency (1158; 41.8%), while college graduates had the lowest frequency (43; 1.6%). Regarding the occupational category, housewives had the highest frequency (1158; 41.8%), while white-collar workers had the lowest frequency (19; 0.7%). Regarding the marital status category, widows had the highest frequency (2173; 78.5%), while singles had the lowest frequency (76; 2.8%). Furthermore, 2260 participants (81.6%) did not receive basic livelihood benefits, 467 participants (16.9%) were receiving basic livelihood benefits, and 39 participants (1.4%) had received basic livelihood benefits in the past at the time of the survey. Regarding the monthly income category at the time of the survey, the 500,000 to 990,000 Korean Won category had the highest frequency (1216; 43.9%) while the 2 million Korean Won and over category had the lowest frequency (186; 6.7%), excluding missing data. Mean scores for participation in social, religious, and philanthropic activities and satisfaction with the physical neighborhood environment, social neighborhood environment, and public services condition were 2.60, 2.48, 5.50, 5.20, and 3.68, respectively. Mean scores for subjective health status and happiness were 2.58 and 6.23, respectively. The skewness ranged from −.77 to .72, and kurtosis ranged from −.67 to 1.8, which indicates that the data met the normal distribution.

**Table 1:** General Characteristics of Subjects (*N* = 2768)

Characteristics	Categories	n	%	M ± SD	Range	Skewness	Kurtosis
Gender	Male	605	21.9				
	Female	2163	78.1				
Age (years)	65–69	682	24.6	74.89 ± 6.43	65–100		
	70–74	701	25.3				
	75–79	719	26.0				
	Over 80	666	24.1				
Education	Illiteracy	502	18.1				
	Elementary school	1158	41.8				
	Middle school	455	16.4				
	High school	434	15.7				
	College	43	1.6				
	University	141	5.1				
	≥Graduate school	26	1.0				
	No response	9	0.3				
Occupation	Housewife	1158	41.8				
	Blue collar	404	14.6				
	Service/marketing	126	4.6				
	Agriculture/Forestry/Fishing	35	1.3				
	Specialized job	29	1.0				
	White collar	19	0.7				
	Not occupied	997	36.0				
Marital status	Widow	2173	78.5				
	Divorced	280	10.1				
	Separated	120	4.3				
	Married	114	4.1				
	Single	76	2.8				
	No response	5	0.2				
Basic livelihood	Yes	467	16.9				
recipient	Not now, but in the past	39	1.4				
	No	2260	81.6				
	No response	2	0.1				
Monthly income (10,000 won^1^)	< 50	924	33.4				
	50–99	1216	43.9				
	100–199	436	15.8				
	≥200	186	6.7				
	No response	6	0.2				
**Exogenous Variables (Total Score)**				**M ± SD**	**Range**	**Skewness**	**Kurtosis**
Participation in social activities (4)				2.60 ± 0.69	2–4	0.72	− 0.67
Participation in religious and philanthropic activities (4)				2.48 ± 0.57	2–4	−0.70	−0.50
Satisfaction with the physical neighborhood environment (6)				5.50 ± 0.84	3–6	−1.65	1.80
Satisfaction with the social neighborhood environment (6)				5.20 ± 0.91	3–6	−0.81	−0.42
Satisfaction with the condition of public services (4)				3.68 ± 0.64	2–4	−1.77	1.72
**Endogenous Variables (Total Score)**				**M ± SD**	**Range**	**Skewness**	**Kurtosis**
Subjective health status (5)				2.58 ± 0.94	1–5	0.14	−0.47
Happiness (10)				6.23 ± 2.10	1–10	−0.10	−0.20

^1^One USD is approximately 1200 Korean won

### Goodness-of-fit test of the hypothesized model

**Fig. 2:**
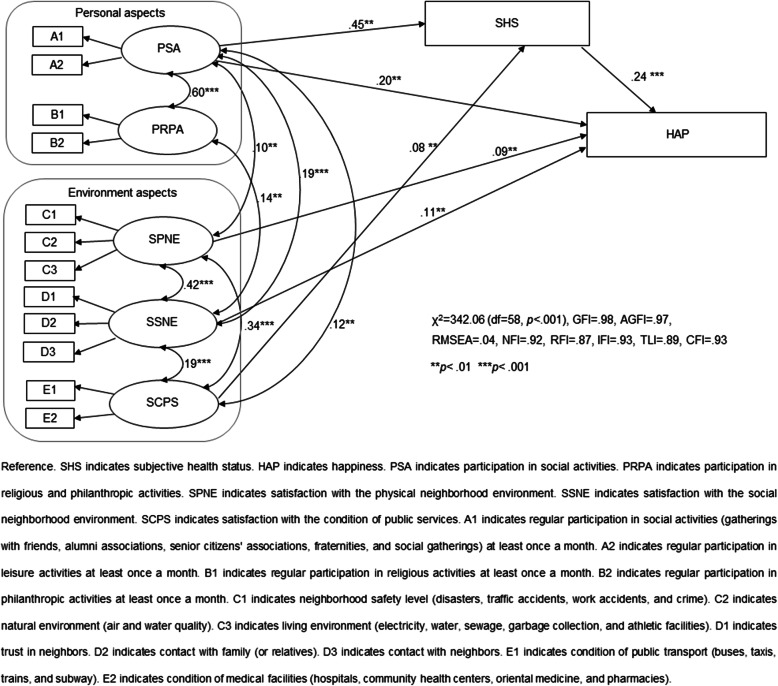
Path diagram for the hypothetical model

As evidenced by the correlation coefficient of <.7, VIF < 10, and tolerance >.1, no multicollinearity existed among the observational variables. The survey tool was deemed to exhibit adequate construct validity based on the Kaiser-Meyer-Olkin measure of .65, the χ^2^ test value of 3261.72, and Bartlett’s squareness test (df = 66, *p* < .001). The observational variables seemed to reflect these latent variables because all standardized factor loads were above .51, and all critical ratio values were significantly above 2.87. The overall structural model for older adults living alone exhibited the following goodness-of-fit indices: χ^2^ = 342.06 (df = 58, *p* < .001), GFI = .98, AGFI = .97, RMSEA = .04, NFI = .92, RFI = .87, IFI = .93, TLI = .89, and CFI = .93. An optimal model (Fig. 2) comprising significant path coefficients was derived, and the model fitness indices were within the recommended levels.

### Research hypothesis testing and effect analysis

Once the validity and reliability of the model were confirmed, the hypotheses were tested. Table 2 presents the path coefficients observed between the theoretical variables derived from the preceding structural analysis. Figure 2 summarizes the model with significant path coefficients.

**Table 2:** Standardized Direct, Indirect, and Total Effects in the Modified Model

Endogenous variables	Exogenous variables	SE	CR	***p***	Parameter Estimate			SMC
					Direct effect(p)	Indirect effect(p)	Total effect(p)	
SHS	PSA	.32	7.10	.001	.45(.001)		.45(.001)	.18
	PRPA	.46	−1.17	.228	−.07(.228)		−.07(.228)	
	SPNE	.17	0.97	.354	.03(.354)		.03(.354)	
	SSNE	.13	−1.01	.352	−.03(.352)		−.03(.352)	
	SCPS	.10	2.91	.008	.08(.008)		.08(.008)	
HAP	PSA	.61	3.80	.003	.20(.003)	.11(<.001)	.31(<.001)	.54
	PRPA	.85	0.70	.565	.04(.565)	−.02(.209)	.02(.734)	
	SPNE	.33	2.87	.008	.09(.008)		.09(.008)	
	SSNE	.25	3.67	.002	.11(.002)		.11(.002)	
	SCPS	.19	0.54	.595	.01(.595)	.02(.006)	.03(.241)	
	SHS	.05	10.11	<.001	.24(<.001)		.24(<.001)	

SHS indicates subjective health status. HAP indicates happiness. PSA indicates participation in social activities. PRPA indicates participation in religious and philanthropic activities. SPNE indicates satisfaction with the physical neighborhood environment. SSNE indicates satisfaction with the social neighborhood environment. SCPS indicates satisfaction with the condition of public services. SMC indicates Squared multiple correlations

The path coefficient for the effect of participation in social activities on the happiness and subjective health status of older adults living alone was .20 (*p* = .003) and .45 (*p* = .001), respectively. The path coefficients for the effect of participants’ satisfaction with the physical and social neighborhood environments on their happiness were .09 (*p* = .008) and .11 (*p* = .002), respectively. The path coefficient for the effect of participants’ satisfaction with the condition of public services on their subjective health status was .08 (*p* = .008). The path coefficient for the effect of participants’ subjective health status on their happiness was .24 (*p* < .001). Regarding the standardized total, direct, and indirect effect values, only participation in social activities increased significantly to .31 (*p* < .001), indicating that participants’ happiness increased through their subjective health status. In this study, participation in social, religious, and philanthropic activities, satisfaction with the physical and social neighborhood environments and the condition of public services, and subjective health status explained about 54.0% of the variance in the happiness of older adults living alone.

## Discussion

This study aimed to develop and test a structural equation model that elucidates the relationship between the subjective health status and happiness of older adults living alone and how their participation in social activities and satisfaction with the neighborhood environment affects this relationship. In this study, 16.9% of the subjects were found to be eligible for basic living. In Korea, basic living beneficiaries are a public welfare system provided to low-income vulnerable groups. In the study of Hwang and Sim [24], it was found that the older adults living alone had more basic livelihood recipients that those living with families. It is indicating the economic vulnerability of older adults living alone than those living with families. Findings revealed that only participation in social activities positively correlated with the subjective health status and happiness of older adults living alone. This result is consistent with Kim and Ha [17] that productive leisure activities, such as exercise, volunteering, and travel, positively affect the physical and mental health of older adults living alone. Likewise, Djundeva and colleagues [5] found that older adults who were co-residing with their peers had higher overall well-being than those with a restricted social network and further emphasized that relationships with family and friends contribute positively to well-being. Lee and colleagues [25] stated that older adults living alone formed social networks through participation in various programs at senior welfare centers and that these activities brought them happiness. Wu and Chan [26] determined that social contact with friends reduced the perceived isolation of older adults living alone in large cities more effectively than contact with relatives who had no connections with them. These same authors demonstrated that the sense of belongingness and emotional support provided by friendships and leisure activities with friends of similar ages and life experiences mitigated the isolation experienced by older adults who engaged in little social interaction. The evoking of healthy feelings led to greater happiness [26]. However, other work showed that older adults living alone were more likely to experience lower levels of belonging with friends and participate in fewer leisure activities than those living with others [27], suggesting that some groups are more vulnerable than others. The present results show that participation in social activities is significantly related to the satisfaction with the socio-physical neighborhood environment, suggesting the importance of the environment in enhancing the health and happiness of older adults living alone. Therefore, the government and community should develop and provide social leisure activities for this population.

The results also show that participation in religious and philanthropic activities does not significantly affect the subjective health status and happiness of older adults living alone. This finding is inconsistent with Lee and colleagues [25] that older adults who live alone only experience happiness by engaging in volunteer work. A literature review by van Leeuwen and colleagues [14] revealed a large variation in the value that older adults placed on faith and religion. For some respondents, religion was not important in their daily life while, for others, religion helped in coping with a disability, psychological distress, and change, and contributed to life satisfaction. In a study of 480 older adults of Christian faith, Kang and Cho [28] considered that religious activities positively impacted the respondent’s satisfaction in life. This satisfaction was not affected by the respondents’ feelings and attitudes toward religious faith.

These results suggest that the religious activities of older adults living alone provide life satisfaction and may further contribute to happiness because family-like intimacy may be formed through frequent exchanges with members of religious groups rather than through common religious values. This inference may be supported by the present results that participation in religious and philanthropic activities was significantly related to satisfaction with the social neighborhood environment and participation in social activities.

The present results indicated a positive association between the happiness of older adults living alone and their satisfaction with their socio-physical neighborhood environments. The findings of Stahl and colleagues [19] supported that the more negative the perception of the social quality of the neighborhood environment, the lower the sense of community, and the higher the association with depression among older adults living alone. Additionally, a sense of solidarity with others, the community, or the neighborhood improves well-being and provides a sense of independence [3]. Further evidence suggests that the support from families, friends, and others contributes to happiness, a sense of coherence, perceived health, and life satisfaction [7, 22, 23]. Therefore, a clean and safe local environment that facilitates social engagement is essential for promoting the happiness of older adults living alone.

Social isolation and loneliness are serious public health problems that affect the physical and mental health of older adults. The present results determined that although satisfaction with the physical and social neighborhoods did not significantly affect the subjective health status of older adults living alone, it significantly affected their happiness. Previous literature supports these findings. Van Leeuwen and colleagues [14] found that older people who experience close intimacy with their neighbors felt safe in their neighborhood. Stahl and colleagues [19] indicated that older adults living alone were more affected by their social neighborhood environment because of the help received from, trust in, and a sense of deep belonging with neighbors. Wu and Chan [26] suggested that daily participation in neighborhood settings and neighborhood events in public apartments positively affected social interactions and substantially reduced the levels of loneliness among older adults living alone. Zebhauser and colleagues [4] reported that older adults who lived alone but had a high social network index experienced noticeably lower levels of loneliness than those with a low social network index. Similarly, Lee and colleagues [25] found that a mutually supportive environment among residents in which residents felt they belonged to a community, engaged in mutual exchange, and felt attachment affected the health-related quality of life of residents. Putrik and colleagues [18] reported that social relations with neighbors and perceived safety of the neighborhood environment had a marked effect on residents’ subjective health status. Therefore, members of a group with strong mutual levels of trust also have a better quality of life than those without, suggesting that satisfaction with the social neighborhood environment affects happiness.

In this study, although satisfaction with the condition of their public services did not significantly affect the happiness of older adults living alone, it had a significant effect on their subjective health status. These results are supported by van Leeuwen and colleagues [14], who found that some environmental barriers, such as poorly designed traffic services, could restrict older adults’ participation in community life. Similarly, Putrik and colleagues [18] reported that when the living environment was safer, older adults’ social connection was higher, and car or subway traffic disorder was lower. Consequently, older adults had better subjective and mental health.

In modern societies, individuals are more likely to be left alone due to increased life expectancy and the death of their spouses, which may make them feel lonely and alienated. The Korean Government has recently started to promote community care so that care individuals who require care can continue to live in their homes rather than in elderly care facilities, enjoy the necessary health and welfare services, live in their communities, and participate in community activities. These policies intend to ensure that those who require care are provided with the same level of care in a familiar home or community rather than in a facility. Older adults living alone could overcome the depression caused by the loss of social roles and loss of support from their families if they receive the necessary support through various social networks and are able to maintain communication with the outside world. The results of the present study demonstrate that the socio-physical environments of older adults living alone and their participation in various social activities could have a positive effect on their health and happiness. Therefore, the government and related organizations should attempt to improve the physical and social neighborhood environments of older adults living alone by considering the characteristics of the neighborhoods and develop and implement appropriate legal and institutional policies. Additionally, they should provide financial support to these individuals to facilitate their participation in various social and leisure activities.

### Limitations

This study had several limitations. There is difficult to make causal inferences, because the secondary data collected were cross-sectional. Attempts to generalize the results of this study, which were obtained using secondary data originally collected for another purpose, must be undertaken with caution. Although this study incorporated a representative population using secondary data obtained from the 2017 KCHS, it only used variables presented in the original data. General, social, and environmental characteristics were analyzed without a standardization process. Happiness is a very broad concept in which several factors work in combination. In other words, there is a limit to measuring the happiness of older adults based on the variables presented in this study. Furthermore, the variables related to the happiness of the participants in this study were very limited. The data used in this study were collected using a structured self-report questionnaire. Thus, the possibility of response bias cannot be eliminated.

### Implications for further research

Therefore, further continuous and repetitive studies will be needed to reveal the variables affecting the happiness of older adults living alone. A study comparing correlates of happiness between those living alone and those living with others would provide meaningful implications to the research area. Moreover, intervention studies to improve the health and happiness of older adults living alone should target the variables identified in the present study as having significant effects on the participants’ health status and happiness.

## Conclusion

This study aimed to develop and test a structural equation model to empirically elucidate the relationship among participation in social activities, satisfaction with the neighborhood environment, subjective health status, and happiness in older adults living alone in Korea. Ultimately, it aimed to provide a comprehensive understanding of the well-being of older adults living alone and create a basis for developing an intervention program to promote a healthy and happy life for this population. The study found that the social problems faced by older adults living alone should not only be attributed to the older adults but suggests the need for active interventions at a social level. These interventions should focus on improving the social and physical environments of older adults living alone. Based on the results, we suggest the following interventions to increase happiness among older adults living alone. First, it is necessary to build a safe, local environment and provide convenient services. Second, social programs with friends should be developed and provided. Therefore, interventions for enhancing the health and happiness of older adults living alone should improve their physical and social environment based on the characteristics of their neighborhoods and participation in various social activities.

## Data Availability

All datasets used and analyzed during the current study are property of the Korea Disease Control and Prevention Agency (KDCA), Chngcheongbuk-do, Republic of Korea. The database if available from the corresponding author on reasonable request.

## Abbreviations

SHS: Subjective Health Status
HAP: Happiness
PSA: Participation in Social Activities
PRPA: Participation in Religious and Philanthropic Activities
SPNE: Satisfaction with the Physical Neighborhood Environment
SSNE: Satisfaction with the Social Neighborhood Environment
SCPS: Satisfaction with the Condition of Public Services
